# Cognitive effects of intravenous Ketamine in treatment-resistant depression: A systematic review

**DOI:** 10.1017/S0033291726103961

**Published:** 2026-05-08

**Authors:** Lou-Anne Chavigny, Véronique Desbeaumes Jodoin, Nicolas Garel, Manola Sob Ndongo, Laura Osborne, Gustavo Turecki, Stéphane Richard Devantoy

**Affiliations:** 1 McGill University, Canada; 2 Hospital Notre Dame: Hôpital Notre-Dame, Canada; 3 CHUM Research Centre: Centre de Recherche du Centre Hospitalier de l’Universite, Canada; 4 Psychiatry, McGill University, Montreal, Quebec, Canada

**Keywords:** cognition, executive function, ketamine, processing speed, suicidal cognition, treatment-resistant depression

## Abstract

Intravenous (IV) low-dose ketamine has emerged as a promising treatment for patients with treatment-resistant depression (TRD). However, its impact on cognitive functioning remains unclear. This systematic review examines the cognitive and executive effects of IV ketamine in TRD, focusing on their relationship to depressive and suicidal outcomes. A systematic search of Cochrane, MEDLINE, Embase, and PsycINFO databases was conducted up to May 15, 2025, using the terms depression, cognition, and ketamine. This review was conducted in accordance with the PRISMA guidelines, and the protocol was registered in PROSPERO (ID: 1160487). Risk of bias was evaluated using the Cochrane RoB 2 tool for randomized trials and the ROBINS-I tool for non-randomized studies. Twenty-one studies, comprising approximately 900–1,180 participants with TRD, assessed cognitive domains of processing speed, working memory, attention, verbal and visual memory, cognitive flexibility, and executive control. Procognitive effects were frequently observed in processing speed and working memory, while attention results were preserved or modestly improved, and verbal and visual memory results were heterogeneous. Executive control, particularly inhibitory performance on Stroop paradigms, improved in several trials. Two studies directly examined cognition as it relates to suicidal behaviors. No cognitive deterioration was reported. Subanesthetic IV ketamine appears to preserve and enhance specific cognitive functions in TRD, notably across processing speed, working memory, and executive control. These procognitive effects, particularly in executive control, may mediate ketamine’s antisuicidal action. Standardized longitudinal studies are warranted to clarify their durability and clinical significance.

## Introduction

Major depressive disorder (MDD) is a leading cause of disability worldwide and a major public health concern (Kennedy et al., 2016). Antidepressant medications targeting monoaminergic systems, as well as evidence-based psychotherapies such as cognitive-behavioral therapy, behavioral activation, and interpersonal therapies, have demonstrated efficacy (Kennedy et al., 2016). However, 30%–40% of patients fail to achieve full remission even after several treatment phases, rendering their depressive episode treatment-resistant (Demyttenaere & Van Duppen, 2019; Nemeroff, 2007). Following two consecutive antidepressant failures, the probability of a patient’s response to a third treatment attempt falls below 10% (Rush et al., 2006; Souery et al., 1999). Treatment-resistant depression (TRD) is associated with marked impairments in global functioning, elevated suicide risk, and persistent cognitive deficits, particularly in memory, attention, processing speed, and executive functions (Rock et al., 2014; Snyder, 2013). These cognitive disturbances often persist despite mood remission and contribute to functional disability and vulnerability to depressive relapse.

A promising non-monoaminergic target is the glutamatergic system. Ketamine, a non-competitive N-methyl-D-aspartate (NMDA) receptor antagonist, first introduced as an anesthetic in the 1970s, has shown rapid antidepressant effects at subanesthetic doses. In 2000, Berman and colleagues reported a robust, rapid (within 24 hours) but transient antidepressant response following a single intravenous (IV) ketamine infusion (0.5 mg/kg over 40 minutes) in seven patients with TRD. This landmark study, since replicated in numerous randomized controlled trials, marked a paradigm shift in the treatment of depression (Marcantoni et al., 2020). Though evidence remains limited, the Berman dosing protocol remains the most widely used and has shown comparable efficacy to electroconvulsive therapy (ECT) in non-psychotic TRD (Jha et al., 2024).

While the antidepressant effects of IV ketamine are well established, its impact on cognitive functioning remains less clearly defined (Gill et al., 2021). Preliminary evidence suggests potential enhancements in processing speed and working memory through glutamatergic and neuroplasticity-related mechanisms (Murrough et al., 2013). The systematic review by Gill et al. (2021) identified improvements in processing speed and verbal memory but did not include studies published in the past 5 years and did not address cognitive processes associated with suicidal behaviors. Since then, several recent trials (Kumpf et al., 2025; Oughli et al., 2023; Permoda-Osip et al., 2015; Phillips et al., 2022; Richard-Devantoy et al., 2014; Zavaliangos-Petropulu et al., 2023; Zhou et al., 2022) have expanded the evidence base by exploring cognition beyond mood improvement, partially including domains relevant to suicidal cognition, such as executive control and verbal fluency (Richard-Devantoy et al., 2014, 2024).

The aim of the present study was therefore to conduct a systematic and critical review of the literature on the cognitive effects of IV ketamine in TRD, with particular attention to executive functions and cognitive processes related to suicidal behaviors.

## Materials and methods

### Search strategy and selection criteria

Cochrane, MEDLINE, Embase, and PsycINFO databases were searched up to May 15, 2025, using the terms ‘major depressive disorder’, ‘treatment-resistant depression’, ‘intravenous ketamine’, ‘cognition’, and their synonyms. Search strategies combined controlled vocabulary (e.g. MeSH, Emtree) and free-text terms. Searches were limited to human studies in adults. To reduce heterogeneity, studies involving esketamine or intranasal ketamine were excluded. Inclusion criteria were as follows: (1) adult participants with treatment-resistant major depressive disorder (TRD); (2) the use of single or multiple IV ketamine infusions; intranasal/esketamine and non-IV routes were excluded; (3) cognition assessed as an outcome; (4) publication in a peer-reviewed journal; and (5) articles published in English or French. This review was conducted in accordance with the PRISMA guidelines, and the protocol was registered in PROSPERO (ID: 1160487).

**Figure 1.**
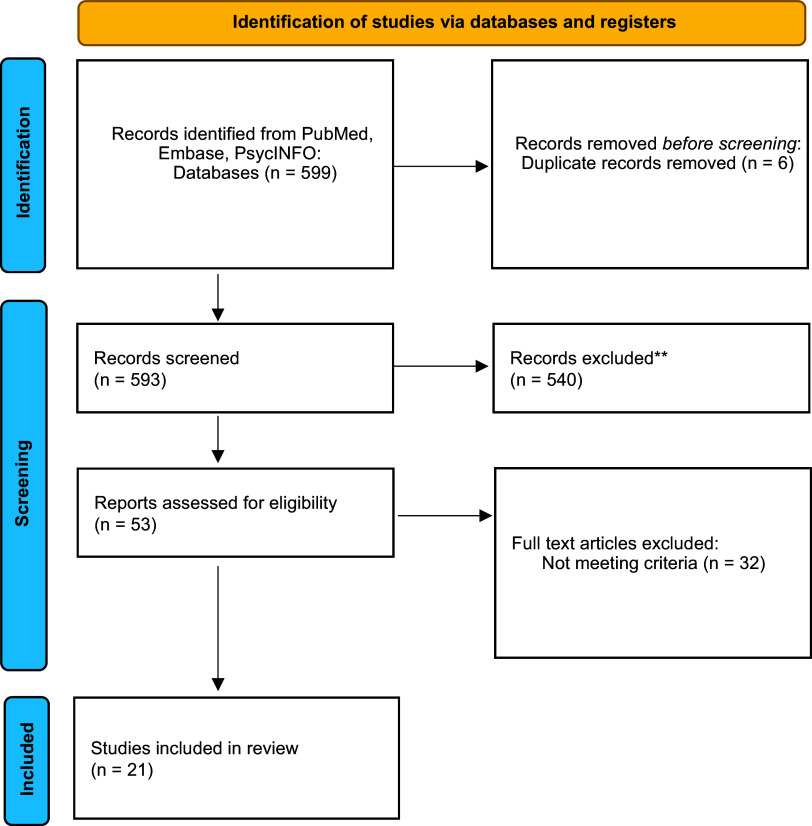
Prisma flow chart for studies identified through databases and registers.

The database search initially identified 599 records. After removal of 6 duplicates, 593 records were screened at the title and abstract level, resulting in 540 exclusions. Fifty-three full-text articles were assessed for eligibility; 32 were excluded primarily for not meeting TRD or IV-ketamine criteria. In total, 21 studies met the inclusion criteria (Figure 1), including several recent trials (Chen et al., 2018, 2022; Jha et al., 2024; Keilp et al., 2021; Kumpf et al., 2025; Liu et al., 2019; McIntyre et al., 2021; Murrough et al., 2015; Oughli et al., 2023; Permoda-Osip et al., 2015; Phillips et al., 2022; Price et al., 2014; Shiroma et al., 2014, 2020; Singh et al., 2024; Zavaliangos-Petropulu et al., 2023; Zheng et al., 2019; Zhou et al., 2022, 2021, 2018). Given the overlap between some research groups, there is a potential for sample overlap across certain studies (Chen et al., 2018, 2022; Zhou et al., 2022, 2021, 2018). Each study was retained when it reported distinct cognitive outcomes, analyses, or time points; however, this may lead to duplications in participant counts.

### Data collection and extraction

Abstracts retrieved through the search were independently screened by three reviewers (LAC, SRD, and VDJ) to determine eligibility. Full texts were then obtained for studies that met the inclusion criteria. For each eligible study, the following data were independently extracted: (1) study characteristics (first author, year, country, eligibility criteria, and sample size per group); (2) participant characteristics (age, sex, and psychiatric diagnoses); (3) ketamine treatment details (dose, frequency, route of administration, and duration); (4) depression measures; (5) cognitive measures (neuropsychological tests, cognitive domains assessed, and timing of assessment); and (6) a summary of the study’s main findings. When multiple publications originated from the same registered trial or clearly shared cohort, identified based on trial registration number, recruitment site, recruitment period, and study design, we designated a primary cohort report for participant counting and overall synthesis. Additional publications from the same cohort were retained when they contributed distinct cognitive domains, mediation analyses, or non-overlapping follow-up intervals not reported in the primary report. This strategy was implemented to enhance methodological transparency and to prevent inflation of the total sample size due to cohort reuse.

### Outcomes

The primary outcome was a change in cognitive performance across domains, assessed using standardized neuropsychological tests. Owing to substantial heterogeneity in study design, ketamine protocols, timing of assessment, and cognitive measures, quantitative meta-analysis was not feasible. Therefore, a structured narrative synthesis using descriptive vote-counting was conducted to summarize the number of studies reporting improvement, no change, or worsening within each cognitive domain.

Although this approach does not yield pooled effect size estimates or formally weight studies by sample size, larger randomized trials were given particular interpretative emphasis in the narrative discussion. This strategy allowed identification of consistent patterns across heterogeneous studies while maintaining transparency regarding methodological variability.

### Assessment of methodological quality

The methodological quality of the included studies was assessed independently by three reviewers using validated tools recommended by the Cochrane Collaboration. Inter-rater agreement between the three reviewers was substantial (Fleiss’ κ = 0.72), indicating a reliable assessment of study eligibility and risk of bias. Randomized controlled trials (RCTs) were evaluated with the Cochrane Risk of Bias 2 (RoB 2) tool, which considers five domains: (1) randomization process, (2) deviations from intended interventions, (3) missing outcome data, (4) measurement of the outcomes, and (5) selection of the reported results. Non-randomized or open-label studies were evaluated with the Risk of Bias in Non-randomized Studies of Interventions (ROBINS-I) tool, which covers seven domains: confounding variables, selection of participants, classification of interventions, deviations from intended interventions, missing data, measurement of outcomes, and selection of the reported results. Discrepancies were resolved through consensus. Given the heterogeneity across study designs, cognitive assessment tools, and timing of post-treatment evaluations, a quantitative meta-analysis was not feasible. Therefore, findings were synthesized narratively, emphasizing patterns of improvement across cognitive domains. Risk-of-bias outcomes for all included studies are summarized in Table 1.

**Table 1.** Risk of bias assessment of included studies (RoB 2 and ROBINS-I)

Study	Design	Tool	D1 Randomization	D2 Deviations	D3 Missing data
Chen et al., 2018	RCT (0.5 vs 0.2 vs placebo)	RoB 2	Low risk	Low risk	Some concerns
Chen et al. 2022	RCT (0.5 vs 0.2 vs saline, BDNF stratification)	RoB 2	Low risk	Low risk	Some concerns
Keilp et al., 2021	RCT (ketamine vs midazolam)	RoB 2	Low risk	Low risk	Low risk
Shiroma et al., 2020	RCT (ketamine vs midazolam)	RoB 2	Low risk	Low risk	Some concerns
Murrough et al., 2015	RCT (ketamine vs midazolam)	RoB 2	Low risk	Low risk	Low risk
Price et al., 2014	RCT (ketamine vs midazolam, suicidality cognition)	RoB 2	Low risk	Low risk	Some concerns (missing suicidality data in some participants)
Phillips, 2022	RCT	RoB 2	Low	Low	Low
Zavaliangos-Petropulu et al., 2023	RCT	RoB 2	Low	Low	Some concerns
Oughli et al., 2023	RCT	RoB 2	Low	Low	Low
Kumpf et al., 2025	RCT (IV ketamine vs ECT, cognition outcomes)	RoB 2	Low risk	Low risk	Some concerns (dropouts in both arms)
Jha et al., 2024	RCT secondary analysis (ELEKT-D, IV ketamine vs ECT, cognition included)	RoB 2	Low risk	Low risk	Some concerns (attrition, especially in the ECT arm)
Singh et al., 2024	RCT (0.5 mg/kg IV, 3 infusions + continuation phase)	RoB 2	Low risk	Low risk	Low risk
Chen et al., 2022	RCT (0.2 vs 0.5 mg/kg IV vs saline)	RoB 2	Low risk	Low risk	Low risk

Study	D4 measurement	D5 Reporting	Overall	Justification	Cohort/Sample overlap
Chen et al., 2018	Some concerns	Low risk	Low risk	Randomized, placebo-controlled; short follow-up; limited cognitive scope.	Independent
Chen et al., 2022	Some concerns (self-report only)	Some concerns	Some concerns	Reanalysis of RCT, strong design but self-report only; selective domains.	Independent
Keilp et al., 2021	Some concerns (practice effects possible)	Low risk	Low risk	Randomized, midazolam control, neurocognitive outcomes well described; limited to 24 h.	Independent
Shiroma et al., 2020	Low risk	Low risk	Low risk	Well-designed RCT, CogState outcomes; moderate sample size.	Independent
Murrough et al., 2015	Some concerns (cognitive measures limited to short-term)	Low risk	Low risk	Well-designed RCT with active control, neurocognitive outcomes assessed; robust but acute follow-up.	Mount Sinai (NY) cohort
Price et al., 2014	Low risk (validated scales, IAT)	Low risk	Low risk	Double-blind RCT; explicit and implicit suicidality measures; small missing data proportion.	Independent
Phillips, 2022	Some concerns	Low	Some concerns	Standard RCT, some concerns in outcome measurement	Independent
Zavaliangos-Petropulu et al. 2023	Low	Low	Some concerns	Longitudinal design, some attrition	UCLA cohort
Oughli et al., 2023	Low	Low	Low	Well-conducted RCT, low risk overall	Older adults (Germany) cohort
Kumpf et al., 2025	Some concerns (limited cognitive battery)	Low risk	Some concerns	Secondary analysis of ELEKT-D RCT; randomization robust, but cognition not the primary outcome, risk of bias from selective measurement.	ELEKT-D multicenter cohort
Jha et al., 2024	Some concerns (MoCA, HVLT only)	Low risk	Some concerns	Well-conducted secondary analysis; cognition not primary endpoint; attrition limits confidence.	Independent
Singh et al., 2024	Low risk	Low risk	Low risk	Well-designed multicenter RCT; standardized RBANS battery used; improvements in immediate/delayed memory, attention, and language; follow-up through continuation phase; low attrition.	Mayo Clinic cohort
Chen et al., 2022	Some concerns (no post-infusion assessment; cognition measured only at baseline)	Low risk	Some concerns	Double-blind RCT, adequate randomization and blinding; minimal attrition; cognitive outcomes limited to baseline predictive analyses rather than longitudinal assessment.	Taiwan cohort (same as Chen 2018 & 2021)

Study	Design	Tool	Overall risk of bias	Bias confounding	Bias selection	Bias classification
Zhou et al., 2018	Open-label, 6 infusions	ROBINS-I	Serious	Serious	Some concerns	Low risk
Shiroma et al., 2014	Open-label, 6 infusions	ROBINS-I	Serious	Serious	Serious	Low risk
Zheng et al., 2019	Open-label, 6 infusions	ROBINS-I	Serious	Serious	Some concerns	Low risk
Zhou et al., 2021	Open-label (6 infusions, older adults)	ROBINS-I	Serious	Serious	Some concerns	Low risk
Permoda-Osip et al., 2015	Open-label	ROBINS-I	Serious	Low	Low	Low
Liu et al., 2019	Open-label	ROBINS-I	Serious	Low	Low	Some concerns
McIntyre et al., 2021	Open-label, retrospective IV ketamine (CRTCE)	ROBINS-I	Not applicable	Serious risk	Some concerns (subset had cognitive data)	Some concerns (DSST, TMT-B only, later removed)
Zhou et al., 2022	Open-label, IV ketamine 6 infusions (suicidal ideation, MCCB)	ROBINS-I	Not applicable	Moderate risk	Some concerns	Low risk (validated battery)

Study	Bias deviations	Bias missing data	Bias measurement	Independent	Bias reporting	Justification
Zhou et al., 2018	Low risk	Some concerns	Some concerns	Independent	Some concerns	No control, open-label; possible confounding; modest sample.
Shiroma et al., 2014	Low risk	Some concerns	Some concerns	Minneapolis VA cohort	Some concerns	Open-label, no control, small sample; confounding by depression severity.
Zheng et al., 2019	Low risk	Some concerns	Some concerns	Guangzhou–Cogstate cohort	Some concerns	Uncontrolled, open-label; expectancy effects likely.
Zhou et al., 2021	Low risk	Some concerns	Some concerns	Guangzhou–MATRICS cohort	Some concerns	Open-label, uncontrolled, older TRD patients; expectancy and practice effects likely.
Permoda-Osip et al., 2015	Moderate	Moderate	Serious	Poznan (Poland) cohort	Some concerns	Single-arm, open-label, small sample
Liu et al., 2019	Moderate	Low	Serious	Independent	Some concerns	Open-label, TRD with anxious vs non-anxious, moderate quality
McIntyre et al., 2021	Low risk	Serious risk	Non-randomized, retrospective design; moderate measurement quality but selective outcome availability.	CANMAT–Toronto cohort	Some concerns	Retrospective, non-randomized study; cognitive data available only for a subset.
Zhou et al., 2022	Low risk	Some concerns	Open-label, but systematic cognitive assessment (MCCB); attrition and absence of control arm limit causal inference.	Guangzhou–MATRICS cohort	Some concerns	Open-label design; cognition assessed systematically using MCCB, but only selected domains reported; attrition and lack of pre-registration limit transparency.

## Results

### Study characteristics

A total of 21 studies were included in the systematic review, encompassing approximately 1,180 reported participants overall (Figure 1). However, accounting for confirmed cohort overlap – most notably across the Zhou et al. (2018, 2021, 2022) publications originating from a single registered trial (ChiCTR-OOC-17012239), related reports from the same Guangzhou research program, and secondary analyses of the ELEKT-D trial – as well as possible overlap within additional single-center programs, the number of unique participants likely ranges between approximately 900 and 1,180. The aggregated sample comprised approximately 900 patients treated with IV ketamine, about 230–250 comparator participants across midazolam, placebo, or ECT conditions, and a small group of healthy controls. Exact totals vary due to differences in study design, sample structure, and partial cohort reuse. A detailed cohort map grouping publications into probable cohort families is provided in Supplementary Table S1.

Across studies, the mean age was approximately 40–41 years, and 55–58% of participants were male. Ketamine was administered intravenously in all studies, most commonly at a subanesthetic dose of 0.5 mg/kg, with a median of six infusions delivered over approximately 12 days. Baseline cognitive evaluation was conducted prior to the first infusion in nearly all trials. Table 2 summarizes the main study characteristics and cognitive outcomes.

**Table 2.** Summary of articles from the systematic literature review studying the association between cognition and Ketamine in treatment resistant depression

Authors	Population	Ketamine				Measures of depression	Cognition			Results	Influence by depression	Executive functions
		IV	Number of treatments	Dosage	Duration of treatment		Domains	Tests	Time of measurement			
(MH. Chen et al., 2018) Taiwan https://doi.org/10.1016/j.jad.2018.07.033	TDM N: 71 47.14% (M) Average age: 47.35 years KET1: N = 24; 12.5% (H) KET2: N = 23; 26.1% (H) MID: N = 24; 37.5% (M)	✓	1	KET1: 0.2 mg/kg KET2: 0.5 mg/kg	Single dose	HDRS	Working memory + attention +	Computerized Working memory and Go No Go Test	-Baseline (i.e. Before the first treatment) −3 days after treatment −14 days after treatment	- Participants receiving 0.5 mg/kg of ketamine showed slight improvements in sustained attention and motor inhibition (measured by the go/no-go task) at day 14. - A significant association was found between changes in depressive symptoms and cognitive performance at day 3 in the 0.5 mg/kg group - No cognitive decline was observed in either group.	Yes	
(MH. Chen et al., 2022) Taiwan DOI: 10.1055/a–1589–6301	TDM N: 71 28.8% (M) Average age: 46.12 years KET1: N = 23; 26.1% (H) KET2: N = 24; 12,5% (H) MID: N = 24; 37.5% (M) HC: N = 23; 39.1% (M)	✓	1	KET1: 0.2 mg/kg KET2: 0.5 mg/kg	Single dose	HDRS	Working memory + attention	Computerized Working Memory and Go No Go Test	-Baseline	- Patients with lower baseline working memory function showed more significant improvement in depressive symptoms after a single 0.5 mg/kg ketamine treatment. - No improvement in sustained attention, as measured by the GoNoGo task. - No post-infusion cognitive change reported.	Yes	
(Chen et al., 2022) Taiwan https://doi.org/10.1007/s00406–020–01221-z	TDM with or without SI N: 96 45.8% (M) Average age: 34.98 years KET+: N = 59; 45.8% (M) KET-: N = 37; 45.9% (M)	✓	6	0.5 mg/kg	12 days	HDRS	Working memory +	MATRICS Consensus cognitive battery MCCB	-Baseline −1 day after the end of treatment −2 weeks after the 6th treatment	- Significant improvement in working memory in the treated group compared to control patients after six ketamine treatments. This improvement is linked to the improvement in depressive symptoms. - A positive correlation between improved working memory and reduced suicidal ideation suggests that treatment improves memory and enables an anti-suicidal ideation response.	Yes	
(Keilp et al., 2021) United States https://doi.org/10.4088/JCP.21m13921	TDM N: 78 39.75% (M) Average age: 38.4 years KET: N = 39; 43.6% (M) MID: N = 39; 35.9% (M)	✓	1	0.5 mg/kg	Single dose	HDRS	Psychomotor speed+ attention cognitive/inhibitory control + working memory + verbal fluency+ memory reaction time+	WAIS-III digit symbol, CPT stroop test/Go-no GO TRT comp COWAT Bushke SRT CRT	−1 to 3 days before the first treatment −24 hours after treatment	- Significant improvements in processing speed, working memory, and inhibitory control, partly independent of mood change. - Stable attention, verbal, and visual memory; no cognitive decline reported.	Yes/no	Cognitive Control/Inhibition
(Liu et al., 2019) China https://doi.org/10.1016/j.jad.2019.08.012	TDM anxious or not N: 50 44% (M) Average age: 38.4 years KET+: N = 30; 43.3% (M) KET-: N = 20; 45% (M)	✓	6	0.5 mg/kg	12 days	MADRS	Processing speed + working memory verbal memory + visual memory	MATRICS consensus cognitive battery (MCCB)	−1 day before the first treatment -One day after last infusion (Day 13) and 2 weeks after last infusion (Day 26)	- Significant improvement in processing speed (Days 13 & 26) and verbal memory (Day 13 only) in the anxious TRD group. - No significant improvement in the non-anxious group. - No correlation between cognitive changes and mood or anxiety symptoms. No cognitive deterioration observed.	No	
(Murrough et al., 2015) United States doi:10.1038/npp.2014.298	TDM N: 62 45% (M) Average age: 46 years KET: N = 43; 43% (M) MID: N = 19; 50% (M)	✓	1	0.5 mg/kg	Single dose	MADRS	Processing speed + working memory verbal memory + visual memory + Reasoning/solving	MATRICS consensus cognitive battery MCCB	-Before the first treatment −1 week after treatment	- One week after treatment (both groups Ketamine and Midazolam), improvements in processing speed, verbal learning, and visual learning. - No effect specific to ketamine or antidepressant response. - No improvement in working memory or reasoning.	No	Reasoning/ problem solving
(Oughli et al., 2023) United States doi:10.1016/j.jagp.2022.11.013	TDM N: 22 47.8% (M) Average age: 71.5 years	✓	8	0.5 mg/kg	Twice weekly for 4 weeks	MADRS	Processing speed + cognitive flexibility + inhibitory control + episodic memory working memory + fluid cognition composite score +	NIH toolbox cognition battery	-Baseline −4 weeks after the last treatment of the acute phase −8 weeks after the last treatment of the	- Improvements in executive functions (working memory, cognitive flexibility, inhibition) and fluid cognition during the acute phase. - No further cognitive gains during the continuation phase; effects not related to depression change. - No cognitive deterioration observed after repeated ketamine infusions.	No	Cognitive Control/Flexibility & Inhibition
(Permoda-Osip et al., 2015) Poland https://doi.org/10.1055/s–0034–1394399	BD N: 18 22% (M) Average Age: 50 years	✓	1	0.5 mg/kg	Single dose	HAMD 17 items	Processing speed + Attention + cognitive flexibility + cognitive inhibition + working Mmmory +	TMT A&B stroop test	-Baseline -The third day after infusion	- Significant improvements in information processing speed, attention, visuo-spatial working memory, and cognitive flexibility 3 days post-infusion.	No	Cognitive Control/Flexibility & Inhibition
(Phillips et al., 2022) Canada https://doi.org/10.1093/ijnp/pyac045	TDM N: 38 45% (M) Average Age: 41.4 years	✓	7	0.5 mg/kg	4–6 weeks	MADRS	Processing speed attention cognitive flexibility + cognitive inhibition + working memory visuospatial memory verbal memory + episodic memory	Stroop test TMT A&B Digit Span CVLT-II RCFT AMI-SF	-Baseline -within the week after the end of the last treatment	- No cognitive decline with ketamine treatment. - Improvements in verbal memory and processing speed; effects largely mood-related except for verbal memory (long-delay recall).	Yes	Cognitive Control/Flexibility
(Price et al., 2014) United States https://doi.org/10.1002/da.22253	TDM N: 57 48% (M) Average Age: 46.2 years KET: N = 36; 14.4% (M) MID: N = 21; 8% (M)	✓	1	0.5 mg/kg	Single dose	MADRS	Implicit suicide cognition +	IAT	-Baseline −24 hours after treatment	- Participants in the ketamine group showed a significant decrease in implicit associations between self and suicidal concepts, as assessed by the IAT. - No change in ‘Death = Me’ associations. - Baseline implicit suicidal associations predicted greater anti-suicidal response to ketamine.	Yes	Decision-related implicit cognition
(Shiroma et al., 2014) United States https://doi.org/10.1016/j.jad.2023.04.015	TDM N: 15 100% (M) Average Age: 52 years	✓	6	0.5 mg/kg	12 days	MADRS	Processing speed attention cognitive flexibility working memory + verbal memory visual memory +	CogState	-Baseline -After the 6 treatments (Day 13)	- Improvements in visual memory and simple and complex working memory after six treatments, not correlated with changes in depressive symptoms. - Patients with poorer attention at baseline were more likely to respond positively to treatment. - No improvement in cognitive flexibility and other cognitive measures.	No	Cognitive Control/Flexibility
(Shiroma et al., 2020) United States https://doi.org/10.1017/s1461145714001011	TDM N: 57 81.4% (M) Average Age: 53 years KET: N = 36; 14.4% (M) 5MID + 1KET: N = 21; 8% (M)	✓	6	0.5 mg/kg	12 days	MADRS	Processing speed + attention cognitive flexibility + working memory + verbal memory visual memory +	CogState	-Baseline -After the 6 treatments (Day 13)	- Significant improvements in processing speed, cognitive flexibility, and working memory after six ketamine treatments, compared to a single infusion. - Visual memory also improved, while verbal memory decreased slightly. - Cognitive changes were independent of depression severity.	No	Cognitive Control/Flexibility
(Zavaliangos-Petropulu et al., 2023) United States https://doi.org/10.1016/j.jad.2023.04.015	TDM N: 66 45.1% (M) Average Age: 39.5 years	✓	4	0.5 mg/kg	2 weeks	HAMD–17 items	Processing speed + attention cognitive flexibility working memory + episodic memory + language	NIHToolbox	-Within 2 weeks before the first treatment -The day after the first treatment -The day after the last treatment −5 weeks after the last treatment	- Improvements in working memory, episodic memory, and processing speed after 1 and 4 ketamine treatments. - Improvements in information processing speed persisted 5 weeks after the last treatment. - Improvements in language, cognitive inhibition, and attention, but did not survive correction for multiple comparisons. - Improvements in working memory, processing speed, and episodic memory were statistically independent of improvements in depressive symptoms.	No	Cognitive Control/Flexibility
(Zheng et al., 2019) China https://doi.org/10.1016/j.jad.2018.12.005	TDM N: 64 39,1% (M) Average Age: 33.3 years	✓	6	0.5 mg/kg	12 days	MADRS	Processing speed + working memory verbal memory + visual memory	MATRICS consensus cognitive battery MCCB	-The day before the first treatment (D0) -The day after the last treatment (D13) −14 days after the last treatment (D26)	- Significant improvement in verbal memory and processing speed after six ketamine treatments (day 13), maintained at day 26. - Improvements remained significant after adjusting for depressive symptoms but were partly mediated by mood improvement (Sobel test). - No significant change in visual memory or working memory.	Partly	
(Zhou et al., 2021) China https://doi.org/10.1016/j.jpsychires.2021.10.037	TDM & BD N: 111 51.4% (M) Average Age: 34.6 years TDM, N = 84; 57.1% (M) BD, N = 27; 33.3% (H)	✓	6	0.5 mg/kg	12 days	MADRS	Processing speed + working memory verbal memory + visual memory	MATRICS consensus cognitive battery MCCB	-The day before the first treatment (D0) -The day after the last treatment (D13) −14 days after the last treatment (D26)	- Significant improvement in verbal memory and processing speed after six ketamine treatments (day 13). - Processing speed improvements were partly independent of mood improvement and remained significant 14 days after the last treatment. - Verbal learning improvements were fully mediated by changes in depressive symptoms. - No improvement in working or visual memory.	Yes	
(Zhou et al., 2018) China https://doi.org/10.1177/0269881118798614	TDM & BD N: 84 47.6% (H) Average Age: 34.8 years TDM: N = 68; 41.2% (M) BD: N = 16; 75% (M)	✓	6	0.5 mg/kg	12 days	MADRS	Processing speed + working memory verbal memory + visual memory	MATRICS consensus cognitive battery MCCB	-The day before the first treatment (D0) -The day after the last treatment (D13) −14 days after the last treatment (D26)	- Significant improvement in verbal memory and information processing speed after six ketamine treatments (Day 13), maintained at day 26 only for processing speed. -Both improvements were fully mediated by changes in depressive symptoms. - No improvement in working or visual memory. - Better baseline visual memory predicted a greater antidepressant response to ketamine.	Yes	
(Zhou et al., 2022) China https://doi.org/10.3389/fpsyt.2022.779326	TDM & BD N: 86 45.3% (H) Average Age: 33.5 years	✓	6	0.5 mg/kg	2 weeks	MADRS	Processing speed + working memory verbal memory + visual memory	MATRICS consensus cognitive battery MCCB	-Baseline -Day 13 (1 day after 6th infusion) -Day 26 (14 days post)	- Significant improvements in processing speed and verbal learning after six ketamine infusions; processing speed improvements persisted at day 26. - Processing speed partially mediated the anti-suicide effect beyond depressive symptom change. - Better baseline processing speed predicted a stronger anti-suicide response and remission.	Partly	
(McIntyre et al., 2021) Canada	TDM N: 68 47.1% (M) Average Age: 44.4 years	✓	4	0.5–0.75 mg/kg	1–2 weeks	MADRS	Processing speed + cognitive flexibility + attention +	DSST TMT-B PDQ-D5	-Baseline -After 2 infusions (Day 7)	- Significant improvements in objective (attention, processing speed and cognitive flexibility) and subjective cognitive measures after repeated ketamine infusions. - Improvements in DSST and PDQ-D5 were mediated by reductions in depressive symptoms, while TMT-B improvements were independent of mood change.	Yes	Cognitive Control/Flexibility
(Kumar Jha et al., 2024) USA doi:10.1001/jamanetworkopen.2024.17786	TDM N: 365 47.7% (M) Average Age: 46 years ECT: 170; 48.8% (M) KET: 195; 46.7% (M)	✓	6	0.5 mg/kg	3 weeks	QIDS-SR–16	Verbal memory +	MoCA HVLT-R NAART–35	-Baseline and at the end of the treatment	- Among patients treated with ketamine, greater improvement was observed in those with moderate-to-severe depression at baseline (vs very severe). -Lower baseline MoCA were associated with greater reductions in depressive symptoms with ketamine. - Baseline premorbid intelligence (NAART–35 < 85) predicted greater antidepressant response to ketamine compared with ECT.	Yes	
(Kumpf et al., 2025) USA	TDM N: 365 47.7% (M) Average Age: 46 years ECT: 170; 48.8% (M) KET: 195; 46.7% (M)	✓	6	0.5 mg/kg	3 weeks	QIDS-SR–16	Processing speed + attention + verbal memory episodic memory cognitive flexibility + verbal fluency speed+ general cognitive functioning	MoCA HVLT-R stroop test COWAT	-Baseline -End of acute phase −6-month follow up	- At the end of treatment, the ketamine group outperformed the ECT group on cognitive tests. - Within-group analyses showed significant improvements in verbal fluency (COWAT) and executive/attentional control (Stroop color reading and interference) after ketamine treatment, with stable memory performance, independent of depression severity. - No significant difference between responders and non-responders to either treatment on the cognitive tests at 6-month follow up.	No	Cognitive Control/Inhibition
(Singh et al., 2024) USA https://doi.org/10.1016/j.psychres.2024.115829	TDM N: 74 37.8% (M) Average Age: 44.4 years	✓	3	0.5 mg/kg	Acute: 11 days continuation: 4 additional weeks	MADRS	Visuospatial + language + attention + immediate memory delayed memory	RBANS	-Baseline -End of acute phase (up to 11 days) -End of continuation phase (week 4)	- Significant improvements in immediate and delayed memory, attention, and language after serial ketamine infusions. - Improvement in immediate memory correlated with depressive symptom reduction, but other cognitive improvements were independent of depression. - Baseline language performance predicted antidepressant response.	Partly	

AMI-SF, Columbia autobiographical memory interview; BD, bipolar disorder; CDRS-R, children’s depression rating; CogState, Cogstate battery; COWAT, controlled oral word association test; CPT, continuous performance test; CRT, choice reaction test; C-SSRS: Columbia-suicide severity rating; CVLT-II, California verbal learning test-II; DSST, digit symbol substitution test; HAMD, Hamilton depression rating scale; HC, healthy controls; HVLT-R, Hopkins verbal learning test revised; IAT, implicit association test; KET, ketamine; PC, patient controls; MADRS, Montgomery-Asberg depression rating scale; MATRICS, measurement and treatment research to improve cognition in schizophrenia; MCCB, MATRICS consensus cognitive battery; M, men; MID, midazolam; MoCA, Montreal cognitive assessment; MMSE, mini-mental state examination; NART-35, North American adult reading test-35; N-Back Task, N-back task; NIHToolbox, NIH toolbox for the assessment of neurological and behavioral function; PDQ-D5, Perceived Deficits Questionnaire - Depression; POMS, profile of mood states; QIDS-SR-16, quick inventory of depressive symptoms – short version; RT, reaction time; RBANS, repeatable battery for the assessment of neuropsychological status; SCWT, stroop color and word test; SRT, simple reaction time; SSI, scale for suicide ideation; TDM, resistant major depression; TMT A et B, trail making test A et B; WAI-II, working alliance inventory – second version; WAIS-II, Wechsler adult intelligence scale.

Most included studies explicitly excluded participants with current alcohol or substance use disorders, frequently requiring negative urine toxicology screening and excluding substance abuse or dependence within the preceding 3–6 months. These exclusions were reported in the majority of randomized controlled trials. One large randomized trial (ELEKT-D secondary analysis) reported inclusion of a small proportion of participants with substance use comorbidity (Kumpf et al., 2025), whereas a small number of studies did not clearly specify substance-related exclusion criteria (Kumar et al., 2024; Permoda-Osip et al., 2015; Price et al., 2014). Overall, the available evidence primarily reflects TRD populations without active substance misuse.

### Timing of baseline cognitive assessment

The timing of pre-treatment cognitive assessment varied slightly across studies. In most trials, cognition was evaluated within 1 week before the first infusion (Chen et al., 2022; Liu et al., 2019; Murrough et al., 2015; Oughli et al., 2023; Shiroma et al., 2014; Zhou et al., 2021). Chen et al. (2018) assessed cognition 2 days prior to infusion, whereas Zheng et al. (2019) and Price et al. (2014) did not specify the exact timing of baseline evaluation.

More recent studies generally performed baseline assessments either on the day of the first infusion (Permoda-Osip et al., 2015; Phillips et al., 2022; Singh et al., 2024), within the week before treatment initiation (Kumpf et al., 2025; Zavaliangos-Petropulu et al., 2023), or prior to randomization (Zhou et al., 2022).

### Timing of post-treatment cognitive assessments

The timing of post-treatment cognitive assessments also varied considerably across studies.

Shiroma et al. (2014) conducted repeated assessments after each infusion (days 3, 5, 8, 10, and 12), while Murrough et al. (2015) evaluated cognition 7 days post-infusion. Chen et al. (2018) included both short-term (day 3) and medium-term (day 14) assessments.

Oughli et al. (2023) used a longitudinal design with evaluations after six infusions across 3 weeks, and again 1 month later, focusing on episodic memory, processing speed, and executive functions. Phillips et al. (2019) performed repeated testing after a single infusion (2 hours, 1 day, and 7 days) to track temporal evolution.

More recent studies extended follow-up windows: Phillips et al. (2022) assessed cognition at day 1, week 1, and week 4, and Zhou et al. (2022) evaluated participants after six infusions and again at 2- and 4-week follow-up. Zavaliangos-Petropulu et al. (2023) measured outcomes at day 3, day 7, and week 4; Permoda-Osip et al. (2015) reassessed participants 3 days after the final infusion. Lastly, Singh et al. (2024) included additional evaluations at the end of the acute phase and during the continuation phase, and Kumpf et al. (2025) compared cognitive performance at weeks 3 and 6 between IV ketamine and ECT treatments.

### Cognitive domains assessed

Across the 21 studies, cognitive domains evaluated included working memory (16 studies, 76.2%), processing speed (15 studies, 71.4%), attention (11 studies, 52.4%), verbal memory (13 studies, 61.9%), visual memory (9 studies, 42.9%), episodic memory (4 studies, 19%), cognitive flexibility (8 studies, 38.1%), executive control (8 studies, 38.1%), and implicit cognition (1 study, 4.8%).

A synthesis of outcomes and the tests performed is presented in Table 2. The most frequent procognitive effects were observed in processing speed and working memory, whereas results for verbal and visual memory were more heterogeneous. In most cases, improvements in working memory and attention correlated with antidepressant response, while gains in processing speed and executive control were often independent of mood change. A detailed synthesis of improvement versus non-improvement across domains is presented in Table 3. For domain-level counts, publications arising from the same cohort were considered as separate reports when they contributed distinct cognitive domains or follow-up intervals. Cohort overlap is detailed in Supplementary Table S1 and should be considered when interpreting frequency counts.

**Table 3.** Cognitive domains improved or not with IV Ketamine in treatment resistant depression

Cognitive domain	Number of studies	Cognition improved	Cognition not improved	% of cognitive improvement										Does cognitive improvement relate to depression improvement?
Speed Processing	15	13	2										87%	No
Working memory	16	9	7						56%					Partly Yes
Verbal memory	13	8	5							61%				Partly Yes
Visual memory	9	4	5					44%						Yes
Episodic memory	4	1	3			25%								Yes
Attention	11	5	6					45%						Yes
Implicit cognition	1	1	0											Yes
*Executive functions (i.e. cognitive control)*														
Cognitive flexibility	8	6	2									75%		Party Yes
Cognitive inhibition	4	4	0										100%	No

#### Processing speed

Across the included studies, processing speed was one of the most consistently improved domains following IV ketamine treatment. Thirteen of 15 studies reported significant post-treatment gains, often emerging after multiple infusions and persisting up to four weeks after the last session (Oughli et al., 2023; Permoda-Osip et al., 2015; Zavaliangos-Petropulu et al., 2023). Recent trials have further strengthened this observation. Zhou et al. (2022) reported significant improvement on MATRICS processing speed tasks, partially independent of mood response. Permoda-Osip et al. (2015) found enhanced performance on the Trail Making Test (TMT) and Stroop speed measures, also unrelated to depressive symptom change. In Singh et al. (2024), immediate memory improvement correlated with depression reduction, whereas gains in attention and language were independent of mood change. Similarly, Chen et al. (2022) reported faster completion times on psychomotor speed tasks.

In comparative studies, Kumpf et al. (2025) demonstrated that processing speed (Digit Symbol Substitution Test, TMT-A) and cognitive flexibility (COWAT, Stroop interference) were better preserved in ketamine-treated patients than in those receiving ECT. These cognitive benefits were largely independent of changes in depressive symptoms. Conversely, Phillips et al. (2022) and a minority of earlier studies did not find significant effects (Murrough et al., 2015).

#### Working memory

Working memory was the most frequently examined cognitive domain, typically assessed using the Digit Span, n-back, or Trail Making Test–B tasks.

Across studies, nine of 16 trials reported significant improvements following ketamine treatment, often in parallel with mood improvement (Chen et al., 2018, 2022; Singh et al., 2024; Zavaliangos-Petropulu et al., 2023; Zhou et al., 2021). In the RBANS-based trial by Singh et al. (2024), both immediate and delayed memory scores improved significantly after three infusions and remained stable during the continuation phase, indicating sustained enhancement in working memory and attention. Similarly, Chen et al. (2022) found improved performance on working memory and attentional control tasks in the higher-dose ketamine group, suggesting dose-dependent facilitation of executive resources. Zhou et al. (2022) also reported significant gains on MATRICS working memory measures correlated with antidepressant response, while Zavaliangos-Petropulu et al. (2023) observed modest but consistent improvements on NIH Toolbox working memory scores. Permoda-Osip et al. (2015) identified discrete gains on complex executive working memory tasks, such as TMT-B and Stroop interference; however, these were unrelated to mood change. In contrast, Phillips et al. (2022) and Murrough et al. (2015) reported no significant effects, whereas Kumpf et al. (2025) reported that working memory was preserved but not improved compared to ECT.

#### Verbal memory

Verbal memory, typically assessed using immediate and delayed recall tasks such as the California Verbal Learning Test (CVLT), Hopkins Verbal Learning Test (HVLT), or story recall measures, was examined in most trials.

Eight studies out of 13 reported significant improvements in verbal memory following repeated ketamine infusions, frequently independent of changes in depressive symptoms (Singh et al., 2024; Zavaliangos-Petropulu et al., 2023; Zhou et al., 2022, 2018). Specifically, Singh et al. (2024) demonstrated robust improvements in immediate and delayed memory as well as language performance after three infusions, with effects persisting during the continuation phase. Zhou et al. (2022) similarly observed gains in verbal learning and recall on MATRICS tasks, while Zavaliangos-Petropulu et al. (2023) noted stable improvement across serial assessments. In contrast, Phillips et al. (2022), Oughli et al. (2023) and Permoda-Osip et al. (2015) found no significant post-treatment change, and Kumpf et al. (2025) reported that although verbal fluency did not increase, it was better preserved in the ketamine group compared with patients receiving ECT.

#### Visual memory

Visual memory, encompassing visuospatial recall, constructional ability, and complex figure learning, was evaluated in several trials. Four studies out of nine reported significant improvement following repeated ketamine infusions, generally after six to seven sessions, with effects largely independent of mood improvement (Shiroma et al., 2014; Singh et al., 2024; Zavaliangos-Petropulu et al., 2023; Zhou et al., 2022). For instance, Zhou et al. (2022) documented enhanced performance on MATRICS visual learning tasks, while Zavaliangos-Petropulu et al. (2023) observed gains on visual episodic recall that persisted up to 4 weeks post-treatment. In the RBANS-based study by Singh et al. (2024), improvements were also noted on visuospatial/constructional indices during the continuation phase, suggesting potential enhancement of visual encoding and retrieval processes with sustained treatment. Conversely, Oughli et al. (2023), Phillips et al. (2022), and Permoda-Osip et al. (2015) did not observe significant post-infusion changes, and Kumpf et al. (2025) found visual memory to be preserved but not improved relative to ECT.

#### Executive functions

Executive functions were evaluated across studies through measures of executive control, inhibitory control, cognitive flexibility, and implicit cognition. Significant improvements following repeated ketamine infusion were reported, particularly on tasks engaging prefrontal regulation and conflict monitoring.

*Inhibitory control.* Four studies out of four reported significant improvement following repeated ketamine infusion. Improvements in Stroop performance were observed within 1–3 days after ketamine infusion, consistent with enhanced interference control (Keilp et al., 2021; Permoda-Osip et al., 2015). Most improvements in inhibitory control occurred independently of changes in depressive symptoms, as reported across several studies (Keilp et al., 2021; Kumpf et al., 2025; Oughli et al., 2023; Permoda-Osip et al., 2015). Phillips et al. (2019, 2022) reported gains on Stroop and Go/No-Go tasks; however, these effects were attenuated after adjusting for mood improvement. Zavaliangos-Petropulu et al. (2023) found reduced interference scores and improved inhibitory control following six infusions, whereas Oughli et al. (2023) reported stable performance in older adults, indicating cognitive safety even in vulnerable populations. Notably, Keilp et al. (2021) demonstrated that improved Stroop performance paralleled the rapid antisuicidal effects of ketamine, supporting a link between inhibitory control and the modulation of cognitions related to suicidal behaviors.

*Cognitive flexibility.* Oughli et al. (2023) and Zavaliangos-Petropulu et al. (2023) both documented significant improvements in set-shifting and mental flexibility tasks (e.g. TMT-B, switching paradigms). Most improvements in cognitive flexibility occurred independently of changes in depressive symptoms, supporting a direct procognitive effect of ketamine. Similarly, Kumpf et al. (2025) reported superior preservation of flexibility and verbal fluency in ketamine-treated patients compared to those receiving ECT, suggesting that ketamine may protect against ECT-related executive slowing.

##### Implicit cognition

Price et al. (2014) uniquely assessed implicit suicidal cognition using the Implicit Association Test (IAT). A significant reduction in maladaptive implicit associations was observed among clinical responders, underscoring ketamine’s potential influence on automatic, non-conscious processes relevant to suicidal risk.

#### Other cognitive outcomes

Attention was generally stable or modestly improved following ketamine treatment. Five studies out of 11 reported significant improvement following repeated ketamine infusions. Zavaliangos-Petropulu et al. (2023) reported stable attentional performance across six infusions using NIH Toolbox measures, while Phillips et al. (2022) and Permoda-Osip et al. (2015) found maintained or slightly improved scores after a single infusion.

Recent data confirmed and extended these findings: Zhou et al. (2022) demonstrated significant gains in sustained attention on MATRICS tasks; Singh et al. (2024) observed parallel improvements in attention and language that persisted during the continuation phase; and Oughli et al. (2023) reported preserved attentional functioning in older adults without evidence of decline. In the ELEKT-D trial, Kumpf et al. (2025) further demonstrated that attention remained stable in ketamine-treated patients, in contrast to the transient attentional slowing observed in those receiving ECT.

#### Risk of bias assessment

The final selection of studies includes several recent trials, which expand the evidence base beyond early proof-of-concept reports. Risk-of-bias assessment indicated predominantly low to some concerns among randomized trials (RoB 2), while non-randomized studies frequently showed serious risk due to confounding (ROBINS-I) (Kumpf et al., 2025; Oughli et al., 2023; Phillips et al., 2022; Permoda-Osip et al., 2015; Singh et al., 2024; Zavaliangos-Petropulu et al., 2023; Zhou et al., 2022). Table 1 provides the full risk-of-bias ratings for each study according to RoB 2 and ROBINS-I criteria. These risk-of-bias considerations further informed the interpretative emphasis placed on larger randomized trials in the narrative synthesis.

## Discussion

This systematic review indicates that IV ketamine administered at subanesthetic doses in patients with TRD is associated with improvements in selected cognitive domains, most consistently in processing speed, working memory, and attention (Table 3). Approximately three quarters of included reports described procognitive effects, (Chen et al., 2018, 2022; Keilp et al., 2021; Kumpf et al., 2025; Liu et al., 2019; Oughli et al., 2023; Permoda-Osip et al., 2015; Price et al., 2014; Shiroma et al., 2014, 2020; Singh et al., 2024; Zavaliangos-Petropulu et al., 2023; Zheng et al., 2019; Zhou et al., 2022, 2021, 2018), whereas the remaining studies reported limited or absent effects (Chen et al., 2022; Murrough et al., 2015; Phillips et al., 2019, 2022).

Among the most replicated findings, working memory and processing speed improved after repeated infusions, with some effects persisting for up to 4 weeks. Gains in working memory were often linked to antidepressant response, while improvements in processing speed appeared partially independent of mood change, suggesting distinct underlying cognitive mechanisms. Ketamine may exert direct neurocognitive effects distinct from its antidepressant action. For example, Chen et al. (2022) reported faster completion times on psychomotor speed tasks, suggesting a potential facilitation of cognitive efficiency even in patients with higher baseline depressive severity. Overall, ketamine appears to enhance processing speed and aspects of executive functioning, with effects that are partly independent of mood change and may reflect direct glutamatergic neuroplasticity. Working memory tends to improve or at least stabilize, particularly after repeated infusions, with effects that often parallel antidepressant response, potentially mediated by glutamatergic modulation of frontoparietal networks.

Effects on verbal and visual memory were more heterogeneous, with some studies reporting mood-independent improvements (Singh et al., 2024; Zhou et al., 2022). Ketamine may enhance or preserve verbal memory and language-related performance, particularly after repeated infusions. However, the magnitude and persistence of these effects remain heterogeneous, likely influenced by differences in cognitive batteries, follow-up duration, and baseline severity of cognitive impairment. Evidence indicates that ketamine may confer modest benefits on visual and visuospatial memory, particularly following repeated or extended treatment. However, findings remain inconsistent across study designs and cognitive batteries. Finally, ketamine does not impair attentional performance and may confer mild procognitive effects, particularly in selective attention domains. These findings reinforce ketamine’s favorable neurocognitive safety relative to traditional antidepressant interventions.

Executive control emerged as a particularly robust cognitive target. Improvements in Stroop performance were observed across several independent cohorts, with some effects detectable within 24 hours of infusion and persisting beyond the acute phase (Keilp et al., 2021; Permoda-Osip et al., 2015; Phillips et al., 2019, 2022; Zavaliangos-Petropulu et al., 2023). Most improvements in executive and inhibitory control appeared independent of mood change, supporting a direct procognitive effect of ketamine. Notably, Keilp et al. (2021) demonstrated that enhanced Stroop performance paralleled ketamine’s rapid antisuicidal effect, supporting the hypothesis that inhibitory control and attentional regulation may mediate its impact on suicidal cognition. Beyond inhibition, cognitive flexibility improved or was better preserved in ketamine-treated patients as well (Kumpf et al., 2025; Oughli et al., 2023). These findings further contribute to evidence for ketamine’s positive impact on prefrontal executive functioning. Taken together, these findings indicate that executive functions, particularly inhibitory control, represent a key cognitive target of ketamine’s effects. Improvements on Stroop paradigms and related measures may not only index frontocingulate neuroplasticity but also mediate rapid reductions in suicidal ideation, highlighting a mechanistic bridge between cognition and clinical response.

Altogether, subanesthetic IV ketamine appears to preserve, and in some cases enhance, neurocognitive functioning in TRD. These results reflect a clinically meaningful outcome for patients with TRD, given the cognitive burden typically observed in this population. Nevertheless, the considerable interindividual variability and short duration of follow-up in most trials limit firm conclusions regarding the durability and functional significance of these effects.

Furthermore, several moderators may influence cognitive outcomes. Treatment characteristics, such as repeated-dose regimens, generally produced stronger effects than single infusions and play a central role in the cognitive analysis (Grunebaum et al., 2018; Phillips et al., 2019). Patient characteristics are also relevant to validity, as younger age and greater baseline cognitive impairment were associated with more pronounced improvement in several studies (X et al., 2023). These findings emphasize the need to incorporate cognitive endpoints into clinical trial design and individualized treatment planning. This variability in timing may contribute to differences in practice effects and test–retest sensitivity across studies.

At a mechanistic level, ketamine’s NMDA receptor antagonism and downstream glutamatergic modulation promote synaptic plasticity, which may underpin its dual antidepressant and procognitive actions (Glue et al., 2024). There is growing interest in sustained-release or combined-modality approaches (e.g. pairing ketamine with neuromodulation or psychotherapy) to prolong these benefits, although interindividual sensitivity and theoretical risks such as excitotoxicity warrant careful monitoring (Luo et al., 2019).

The complexity of TRD is further compounded by psychiatric and medical comorbidities. Because most included trials excluded individuals with active substance use disorders, the generalizability of the present findings to TRD populations with comorbid addiction remains uncertain. Given the known effects of substance use on glutamatergic and executive networks, future research should directly examine whether substance-related neurobiological alterations moderate ketamine’s cognitive effects. Substance use disorders, for instance, may alter glutamatergic and dopaminergic circuits, attenuating ketamine’s cognitive effects, while metabolic or vascular conditions could exacerbate cognitive vulnerability through inflammatory (Zhou et al., 2019) or cerebrovascular pathways (Haroon et al., 2012). Such interactions remain insufficiently characterized and should be systematically evaluated in future studies.

This review has several limitations. Substantial heterogeneity in study designs, ketamine protocols, assessment timing, and cognitive measures prevented quantitative meta-analysis, and findings were therefore synthesized using a narrative vote-counting approach, which precludes formal effect-size estimation. The search was limited to English- and French-language publications, which may have excluded relevant studies. In addition, inclusion was restricted to TRD populations. While this decision enhances clinical relevance, as IV ketamine is primarily used in later-line treatment settings, findings may not generalize to individuals with non-resistant MDD. Partial cohort overlap is also possible, as several reports originated from the same research groups (Chen et al., 2018, 2022; Singh et al., 2024; Zhou et al., 2022, 2021, 2018), and the total sample size should therefore be interpreted as an approximation of unique participants. Practice effects may have contributed to apparent cognitive gains, although some trials attempted to mitigate this through alternate test forms or extended follow-up (e.g. Singh et al., 2024). Finally, several cognitively and clinically relevant domains, such as decision-making, verbal fluency, and suicidal cognition, remain underexplored, despite their importance for functional recovery and relapse prevention.

## Conclusion

This systematic review suggests that IV ketamine administered at subanesthetic doses is associated with procognitive effects in patients with TRD, most consistently in processing speed, working memory, and attention, without evidence of cognitive deterioration. However, cognitions related to suicide behaviors, such as decision-making and verbal fluency, remain underexplored, despite their potential relevance for functional recovery and relapse prevention. Executive functions, particularly inhibitory control as assessed through Stroop paradigms, emerged as promising cognitive targets that may mediate ketamine’s rapid antisuicidal effects. To our knowledge, this is the first systematic review to explicitly highlight suicide-related cognitions, a clinically critical yet neglected dimension within the context of ketamine treatment.

Future studies should incorporate standardized executive-function endpoints, longitudinal designs, and multi-timepoint cognitive assessments to determine the durability, mechanisms, and functional significance of ketamine’s cognitive effects. A better understanding of these cognitive pathways may ultimately guide personalized interventions and improve suicide prevention strategies in TRD.

## Supporting information

10.1017/S0033291726103961.sm001 Chavigny et al. supplementary material Chavigny et al. supplementary material
